# Therapeutic Efficacy of Anti-Interleukin-5 Monoclonal Antibodies in Kimura Disease: A Systematic Review

**DOI:** 10.7759/cureus.110775

**Published:** 2026-06-13

**Authors:** Sylwia Olizarowicz, Michal Mierzejewski, Michal Majewski, Paulina Szczepanska

**Affiliations:** 1 Faculty of Medicine, Medical University of Warsaw, Warsaw, POL; 2 Department of Dentistry, Masovian Dental Center, Warsaw, POL

**Keywords:** benralizumab, eosinophilia, interleukin 5, kimura disease, mepolizumab

## Abstract

Kimura disease (KD) is a rare, chronic inflammatory disorder characterized by painless subcutaneous tumors and lymphadenopathy, predominantly affecting the head and neck region. Its pathogenesis involves a dysregulated T-helper 2 (Th2) immune response leading to lymphoid hyperplasia and eosinophilic infiltration. As interleukin-5 (IL-5) is the cytokine responsible for eosinophil proliferation and survival, anti-IL-5 therapy may be biologically justified in KD. This study aims to investigate the efficacy of benralizumab, reslizumab, and mepolizumab in the treatment of KD. A systematic review following PRISMA guidelines was performed. Although a comprehensive literature search was performed, no published studies or case reports evaluating reslizumab in KD were identified. Nine case reports (11 patients, mean age 36.5 years, one patient received both agents sequentially) were studied regarding benralizumab and mepolizumab. Data extracted included the used agent, previous and concurrent therapy, efficacy, adverse effects, and recurrence. Mepolizumab showed a therapeutic response in seven out of nine cases, including three complete and four partial responses. Benralizumab showed clinical benefit in all treated patients, although one required salvage surgery. However, these findings must be interpreted with caution due to the limited sample size. Clinical improvement enabled the tapering or discontinuation of concomitant immunosuppressing therapy in most responders. No drug-related adverse events were reported over mean treatment durations of 16 months (median 14 months) for mepolizumab and 23 months (median 12 months) for benralizumab. However, cases included in this study may underreport potential long-term or rare adverse effects. Anti-IL-5 agents offer a potential treatment strategy for refractory or recurrent KD, demonstrating efficacy in tumor reduction and a favorable safety profile. These biologics are a corticosteroid-sparing alternative for resistant cases. Current evidence is limited by the reliance on small, heterogeneous case series. Multicenter prospective registries or international collaborative cohorts are required to validate these findings and standardize dosing regimens.

## Introduction and background

Kimura disease (KD) is a rare, chronic, and slowly progressive inflammatory disorder that predominantly affects young Asian men in the third to sixth decades of life [1]. However, the condition is not exclusive to this demographic. In Western countries, such as the United States, clinicopathologic studies have revealed a heterogeneous patient profile, with a significant proportion of cases diagnosed in White patients and African American patients [2]. The main symptoms of the disease are painless tumors, commonly localized in the head and neck regions such as the preauricular, submandibular, and cervical areas [1]. Lesions typically range from 1.2 to 6.5 cm in maximum dimension, with an average presenting diameter of approximately 2.3 to 2.8 cm. Over time, as the chronic inflammatory cascade persists, these lesions demonstrate progressive stromal and perivenular sclerosis, which can eventually transform the nodules into fibrotic masses [2]. These tumors are frequently accompanied by regional lymphadenopathy and salivary gland involvement. Although typically painless and lacking obstructive symptoms, their progressive enlargement leads to significant cosmetic disfigurement, which is often the primary reason for seeking medical attention. The chronic nature of the disease and its high recurrence rate can severely reduce the patient's quality of life [2]. While the disease is generally benign, it is occasionally associated with systemic manifestations. Although typically restricted to the head and neck region, rare instances of multifocal involvement have been documented where lesions appear in anatomically distant sites. Approximately 20% of patients with KD develop renal disease. Among this specific cohort of patients with renal involvement, 60-80% present with nephrotic syndrome. The predominant pathology in these cases is membranous nephropathy. Renal biopsies typically reveal subepithelial electron-dense deposits, alongside granular deposits of immunoglobulin G (IgG) and C3 along the glomerular basement membrane. The identification of renal involvement, specifically membranous nephropathy, directly influences treatment selection by prioritizing immunosuppressive therapy. Regimens incorporating corticosteroids, such as prednisolone, alongside calcineurin inhibitors like cyclosporine, have been beneficial, enabling tapering of the dose and sustaining complete remission. Furthermore, although autoantibodies against podocyte antigens, such as the phospholipase A2 receptor (PLA2R), have been implicated in the pathogenesis of this nephropathy, the evidence supporting this association remains limited, and the precise mechanisms are yet to be fully established [3]. A recent case reported a patient with successive lesions involving the orbit, bilateral cubital fossae, and groin, indicating the potential for widespread clinical presentation [4].

The differential diagnosis of KD is extensive and includes both neoplastic and non-neoplastic entities that share clinical and histopathologic features. Epithelioid hemangioma (EH) is the closest mimic, often presenting similarly as pruritic subcutaneous masses in the head and neck. However, unlike KD, EH is characterized histologically by vessels lined with epithelioid endothelial cells that frequently exhibit FosB nuclear positivity. Distinguishing KD from malignancies is important, particularly nodal T follicular helper cell lymphoma and Hodgkin lymphoma, which can be excluded by the absence of neoplastic T-cells or Hodgkin-Reed-Sternberg cells. Langerhans cell histiocytosis is another consideration, differentiated by the presence of histiocytes with grooved nuclei that are immunoreactive for CD1a and CD207, and frequently harbor BRAF V600E mutations. While KD shares features with IgG4-related disease, it lacks the dense IgG4-positive plasma cell infiltrate (typically >10 per high-power field) and the characteristic storiform fibrosis seen in IgG4-related disease [5].

The etiology of KD is unclear, but it is hypothesized to stem from a dysregulated immune response to an unknown trigger, potentially involving self-antigen exposure. This pathogenesis is characterized by a dominant T-helper 2 (Th2) lymphocyte response, where the secretion of interleukin-4 (IL-4), interleukin-5 (IL-5), and interleukin-13 (IL-13) facilitates immunoglobulin class switching to immunoglobulin E (IgE) and stimulates eosinophil recruitment. Recent molecular studies have also implicated overactivation of the Erk/MAPK signaling pathway in eosinophils as a contributing factor. These immunological mechanisms manifest clinically as the disease's characteristic features: painless subcutaneous masses, peripheral blood eosinophilia, and elevated serum IgE levels. Serum IgE is elevated in greater than 90% of cases, often reaching levels many times higher than the upper limit of normal, accompanied by moderate to marked peripheral eosinophilia [5].

Due to overlapping features with both benign and malignant entities, KD presents a diagnostic challenge for which no formal clinical criteria currently exist. However, recent studies have introduced non-invasive tools, specifically a stepwise decision tree model utilizing magnetic resonance imaging (MRI) features. By analyzing quantitative apparent diffusion coefficients (ADCs), lesion location, and lymphadenopathy, this algorithm can accurately differentiate KD from conditions such as lymphoma. Key supporting features identified by this model include para-lesion skin thickening and a unique ADC profile where lymph nodes exhibit lower values than the primary lesion [6]. Although these imaging markers significantly aid the differential diagnosis, biopsy remains essential for final confirmation [5].

Histological examination of the involved lymph nodes consistently reveals preserved nodal architecture with extensive follicular hyperplasia and reactive germinal centers [7]. A prominent eosinophilic infiltrate is a constant feature, typically observed in the interfollicular areas, sinusoids, and perinodal soft tissue. These infiltrates often form eosinophilic microabscesses or show evidence of eosinophilic folliculolysis. Another characteristic finding is the proliferation of postcapillary venules, which may be associated with vascularization of the germinal centers and perivenular sclerosis. Warthin-Finkeldey-type polykaryocytes are frequently identified within the germinal centers or paracortex [5,8].

There is no established treatment strategy for KD, and therapy is mainly symptomatic. Although surgical excision is considered the first-line therapeutic approach, particularly for localized lesions, complete resection is difficult to achieve due to the infiltrative nature of the lesions and the absence of a distinct capsule, leading to postoperative recurrence rates ranging from 25% to 51.7%. A meta-analysis confirmed an overall recurrence rate of 30.5% for surgical excision alone. Systemic corticosteroids are the first-line medical management, often employed as adjuvant therapy to surgery for cases with renal involvement. However, management is frequently complicated by metabolic and cosmetic adverse effects associated with long-term corticosteroid use. Tapering or discontinuing corticosteroid therapy is associated with relapse rates up to 45.8%. Although conventional immunosuppressive agents, such as cyclosporine, have shown efficacy in refractory cases, their long-term application is limited by toxicity profiles. The limitations of conventional therapy (surgery with adjuvant therapy) have led to the investigation of therapeutic alternatives such as biological agents [9,10].

Biological therapy is a potential treatment option for patients with refractory or recurrent disease. Agents such as dupilumab, a monoclonal antibody inhibiting IL-4 and IL-13 signaling, and omalizumab, which targets free IgE, have shown efficacy in reducing tumor burden and controlling pruritus by blocking specific targets of the Th2 inflammatory pathway. These biologics have demonstrated efficacy in cases resistant to first-line corticosteroid therapy and other immunosuppressants. For instance, dupilumab treatment led to the complete resolution of cutaneous nodules and plaques after six months of therapy in a patient with bilateral disease [10]. Similarly, omalizumab induced regression of lymphadenopathy and lesion softening in a patient who had previously failed prednisone and rituximab therapy, with MRI confirming a 20% reduction in mass size following treatment courses [11]. The central role of the eosinophil in the pathophysiology of KD suggests that a more direct approach should be considered. Given that IL-5 is the primary cytokine responsible for the differentiation, recruitment, activation, and survival of eosinophils, therapeutic blockade of the IL-5 axis is a targeted approach [12]. Mepolizumab, reslizumab, and benralizumab are humanized monoclonal antibodies developed to target the IL-5 pathway [13]. However, compensatory cytokine networks may bypass IL-5 blockade. These mechanisms include granulocyte-macrophage colony-stimulating factor (GM-CSF), alpha subunit of the IL-4 receptor, and CCL3/CCR1 axis [12]. Due to this limitation, the clinical utility of the anti-IL-5 strategy requires further investigation.

The objective of this systematic review is to evaluate the efficacy of anti-IL-5 biologics, including mepolizumab, benralizumab, and reslizumab, in the treatment of KD. The study analyzes data on tumor regression and the potential for corticosteroid reduction, as well as safety and dosing requirements. This review evaluates the role of these agents in clinical practice and identifies current research gaps.

## Review

Materials and methods

**Figure 1:**
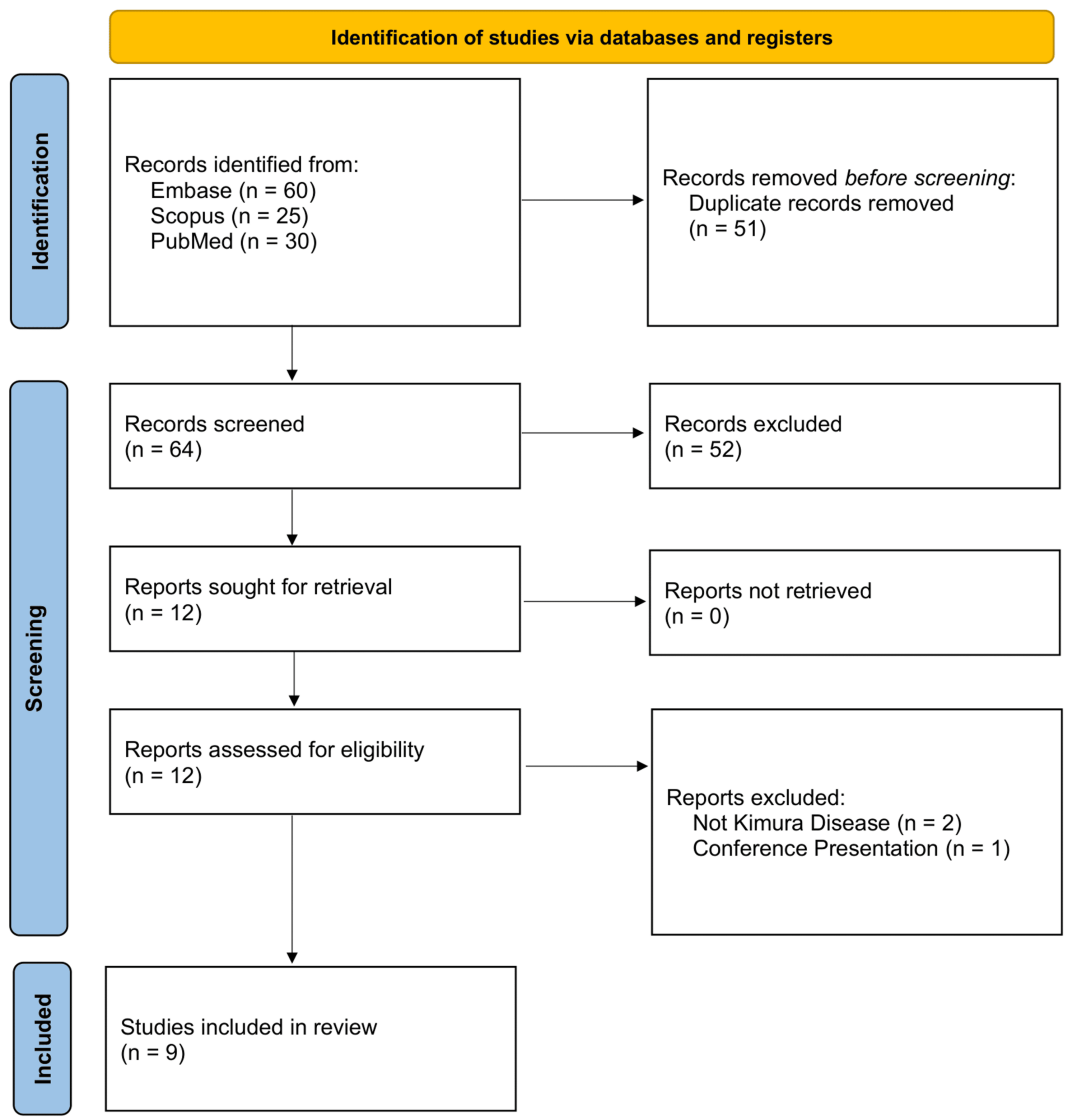
PRISMA 2020 flow diagram depicting the selection process of studies included in the review

This systematic review was conducted in accordance with the Preferred Reporting Items for Systematic Reviews and Meta-Analyses (PRISMA) 2020 guidelines [14]. A comprehensive database search was performed on January 30, 2026, using Scopus, EMBASE, and PubMed databases. A complete search strategy for each database is presented in Appendix 1. The search query ("Kimura Disease" OR "Kimura's" OR "Kimura") AND ("Mepolizumab" OR "Reslizumab" OR "Benralizumab" OR "Anti-IL5" OR "IL-5 receptor") yielded 115 results, of which 51 were duplicates. Screening titles and abstracts revealed 12 eligible studies, and all were later retrieved. Nine studies met the final inclusion criteria for data review. The PRISMA flow chart is presented in Figure 1. All screening steps were undertaken independently by four reviewers. Although formal statistical tools were not used to assess inter-reviewer agreement, a high level of agreement was observed. All disagreements were resolved by consensus to ensure methodological transparency. The PICO framework (Population, Intervention, Comparator, Outcome) defined the population as patients diagnosed with KD treated with benralizumab, mepolizumab, or reslizumab. The off-label intervention of anti-IL-5 agents was predominantly performed after a failure of standard therapy, although two reports included patients treated with biologics as a first-line intervention. Studies not focusing on KD but instead on other hypereosinophilic diseases were excluded, as well as secondary data sources and in vitro experiments. Reports presenting efficacy of other biological agents (e.g., dupilumab) instead of anti-IL-5 drugs were not included. Conference abstracts were also excluded from the final assessment. The methodological quality of the included studies was appraised using the appropriate Joanna Briggs Institute (JBI) Critical Appraisal tools. The risk-of-bias assessment for case reports and the evaluation for case series are presented in Appendices 2 and 3 [15,16].

Extracted data consisted of study characteristics (author, year, country, design), patient demographics (sex, age of onset), intervention specifics (agent, dose, duration, failed treatment, concurrent treatment), investigation findings (baseline eosinophil count, lesion location), and clinical outcomes (adverse effects, efficacy, recurrence, duration of follow-up). Missing data was labeled as not given, and its absence was taken into consideration during data interpretation. Clinical outcomes were stratified into complete response (CR), partial response (PR), and suboptimal response (SR). CR was defined as the achievement of clinical remission characterized by the complete resolution of inflammatory subcutaneous masses and lymphadenopathy, although inactive residual fibrosis was permissible. Classification of CR required the normalization of peripheral blood eosinophil counts and the successful discontinuation of systemic corticosteroids without disease recurrence. PR was defined as a clinically meaningful improvement that did not meet the criteria for remission. This category included patients demonstrating a reduction in lesion size or symptom severity (e.g., pruritus) but with persistent palpable disease activity or incomplete normalization of inflammatory markers. Patients requiring maintenance corticosteroid therapy to suppress symptoms, even at reduced dosages, were strictly categorized as PR. SR denoted a lack of therapeutic benefit, showing by stable or progressive disease, absence of objective tumor regression, or the necessity to switch biological agents due to primary treatment failure. These response categories were initially predefined in the review protocol but were subsequently adapted during the data extraction phase to prevent the misclassification of non-inflammatory scar tissue as active disease, ensuring a more accurate estimation of therapeutic efficacy. Due to the heterogeneity of the included case reports, the absence of control groups, and the sample size limitations within the benralizumab cohort (n = 3), a meta-analysis was not feasible, and a qualitative narrative synthesis was performed to analyze the efficacy of anti-IL-5 agents.

Results

A total of nine studies, including 11 patients (eight male patients, three female patients), met the inclusion criteria and were analyzed in this review [17-25]. The mean age of the cohort was 36.5 years, with a range from 21 to 60 years. Therapeutic interventions were limited to mepolizumab or benralizumab. Although reslizumab is an established anti-IL-5 agent, no studies using this drug were identified in the analyzed cohort. A total of 12 treatment courses were analyzed across 11 patients, as one individual received both anti-IL-5 agents (mepolizumab and benralizumab).

Mepolizumab was the most frequently prescribed agent, administered in nine treatment courses with dosages ranging from 100 mg to 300 mg subcutaneously every four weeks. A therapeutic response was achieved in seven out of nine cases (7/9, 78%), comprising three CR (3/9, 33%) and four PR (4/9, 44%), while two patients experienced SR (2/9, 22%) [17-23]. One patient exhibited a secondary loss of response to mepolizumab characterized by the emergence of lymphadenopathy despite initial improvement, requiring a switch to benralizumab [23]. Benralizumab was employed mostly in three treatment courses of 30 mg every four to eight weeks, with all three treated patients achieving clinical improvement (3/3, 100%), including one CR (1/3, 33%) and two PR (2/3, 66%). Although one patient required salvage surgery to achieve PR, this outcome was classified as a PR rather than an SR, as the patient’s clinical status did not deteriorate following the surgical intervention. The patient achieved stable remission with the continued administration of benralizumab [23-25]. Treatment duration varied widely across the cohort, extending from three months to as long as four years, with a mean duration of 16 months (median 14 months) for mepolizumab and 23 months for benralizumab (median 12 months) [17-25]. While most of the cohort received biologics due to relapsing or refractory disease, two patients received anti-IL-5 agents as a first-line systemic intervention. This approach was chosen to directly target eosinophilia and to avoid the adverse effects of corticosteroid therapy [21,25]. Most patients showing therapeutic responses were able to taper or discontinue concomitant corticosteroids and immunosuppressants such as prednisone and cyclophosphamide. In the cohort of eight patients treated with corticosteroids, seven patients achieved complete corticosteroid discontinuation (7/8, 88%) [17-22,24]. A single case required ongoing corticosteroid therapy without dose reduction. Both agents were well tolerated, with no specific drug-related adverse events reported in the analyzed studies. The development of cervical lymphadenopathy observed in one patient was attributed to disease breakthrough rather than drug toxicity [17]. Due to the limited sample size of the benralizumab cohort, findings regarding the comparative efficacy between mepolizumab and benralizumab should be interpreted with caution. Studies included in the review are presented in Table 1.

**Table 1:** Study characteristics and clinical outcomes of anti-IL-5 therapy in Kimura disease KD: Kimura disease; SC: subcutaneous; TORS: transoral robotic surgery; NG: not given; EGPA: eosinophilic granulomatosis with polyangiitis; G/L: 10^9^/L.

Author	Year	Country	Design	Sex	Age of onset (years)	Age (years)	Agent	Dose	Duration	Failed treatment	Concurrent treatment	Baseline eosinophil counts (G/L)	Lesion location	Duration of follow-up	Adverse effects	Efficacy	Notes	Recurrence
Ho et al. [17]	2021	Australia	Case report	Male	26	26	Mepolizumab	100 mg → 200 mg → 300 mg SC monthly	28 months	Oral and nasal corticosteroids; septal and sinus surgery	Methotrexate	7.65	Upper airway (adenoid tonsils, nasal septum, middle turbinate, lateral wall, frontal sinuses)	28 months	NG	Partial response	Significant improvement; suppression of blood eosinophil count; reduced lymphadenopathy	Developed cervical lymphadenopathy 8 months into treatment on 100 mg dose
Kinoshita et al. [18]	2021	Japan	Case report	Female	36	42	Mepolizumab	300 mg SC monthly	8 months + 6 months after surgery	Surgical resection; oral prednisolone	None	6.30	Both upper limbs	8 months	NG	Partial response	Dramatic regression of masses; eosinophil reduction	NG
Al Shammari et al. [19]	2019	Saudi Arabia	Case report	Male	27	27	Mepolizumab	100 mg SC monthly	3 months	Prednisolone; mesalamine	Azathioprine; mesalamine	3.20	Submandibular and cervical lymph nodes	3 months	NG	Complete response	Remission of symptoms (no neck/lip swelling)	NG
Tao et al. [20]	2025	China	Case report	Male	21	21	Mepolizumab	300 mg SC monthly	3 months	Oral corticosteroids	Oral corticosteroids	3.80	Thyroid and parotid regions	6 months	No	Suboptimal response	Partial improvement of eosinophils but no significant reduction in lymphadenopathy	NG (switched to dupilumab)
Zhu et al. [21]	2025	China	Case series	Male	30	45	Mepolizumab	100 mg SC monthly	24 months	Surgical excision; prednisone	Prednisone (tapered off)	2.42	Multiple masses, lymphadenopathy (submandibular, inguinal, and cervical lymph nodes)	24 months	NG	Complete response	Mass regression; symptom control; corticosteroid discontinuation; low dose due to financial constraints	No
				Male	30 (skin)	39	Mepolizumab	200 mg SC monthly	8 months	Surgical excision; prednisone	Prednisone (tapered off)	3.45	Lymphadenopathy (bilateral axillary lymph nodes)	8 months	NG	Complete response	Disease well controlled; corticosteroid discontinuation; low dose due to financial constraints	NG
					36 (lymphadenopathy)													
				Female	39	43	Mepolizumab	300 mg SC monthly	5 months	None for KD (inhaled corticosteroids and long-acting β2-agonists for asthma)	None	2.83	Lymphadenopathy (left inguinal lymph nodes)	5 months	NG	Partial response	Mass regression; marked improvement in pruritus	NG
Du et al. [22]	2025	China	Case report	Male	30	31	Mepolizumab	300 mg SC monthly	23 months	Prednisone	Prednisone (tapered off)	3.06	Left supraclavicular and bilateral inguinal lymph nodes	26 months	NG	Partial response	Resolution of asthma and rash, but persistent bilateral inguinal lymphadenopathy confirmed as KD by repeat biopsy at 15 months	Enlarged inguinal lymph nodes at 15-month follow-up
Yoon et al. [23]	2025	USA/UK	Case report	Male	27	26 (mepolizumab)	1. Mepolizumab; 2. Benralizumab	Doses NG	Mepolizumab: approx. 3 years; benralizumab: 4 years	Mepolizumab (failed due to disease progression)	Methotrexate (during mepolizumab)	6.50	Cervical lymph nodes, Waldeyer ring, lower extremities (purpuric rash)	96 months	NG	Suboptimal response (mepolizumab); partial response (benralizumab)	Dual diagnosis of KD and EGPA. Mepolizumab was initially started at age 26 for EGPA-related sinusitis. KD (cervical lymphadenopathy) developed at age 27 despite ongoing mepolizumab therapy. Switched to benralizumab at 30 but required urgent TORS and balloon dilation for severe airway obstruction.	Progression of KD on mepolizumab; severe airway narrowing during benralizumab therapy requiring surgical intervention
						30 (benralizumab)												
Szeto et al. [24]	2022	Canada	Case report	Female	39	41	Benralizumab	30 mg SC monthly then every 2 months	9 months	Prednisone; azathioprine; cyclophosphamide	None (weaned off prednisone and cyclophosphamide)	2.90	Cervical lymphadenopathy, skin (maculopapular pruritic and ulcerating rash on arm and leg)	9 months	NG	Complete response	Skin lesions improved; lymphadenopathy resolved; eosinophils undetectable	NG
Talmon et al. [25]	2025	Israel	Prospective study	Male	60	60	Benralizumab	30 mg SC monthly	12 months	None for KD (biologic used as first-line systemic therapy)	None	1.30	Submandibular lymphadenopathy, skin nodules and rash	52 weeks	NG	Partial response	Decrease in size of subcutaneous nodules; complete resolution of rash	Exacerbation occurred before the last dose due to a missed injection

Discussion

Standard management includes surgical excision, radiotherapy, and corticosteroids, yet local recurrence is common. In a meta-analysis of 570 patients, surgical excision alone had a 4.72 times higher recurrence risk than surgery combined with radiotherapy. This high recurrence rate results from the indistinct boundaries of the lesions, which make complete resection difficult. Radiotherapy is primarily indicated as an adjuvant postoperative treatment to control residual microscopic disease and prevent regrowth. It is also considered a viable option for patients with recurrent disease or those who are poor candidates for surgery. Although corticosteroids are frequently used, relapses often occur during dose tapering [9,26].

Mechanism of action

IL-5 serves as the primary cytokine responsible for the differentiation and survival of eosinophils. Its specific action is mediated through a receptor complex consisting of an IL-5-specific alpha subunit and a common beta subunit, which is shared with receptors for other cytokines. IL-5 binding recruits the beta subunit, triggering intracellular signaling cascades. The key mechanism involves the JAK-STAT pathway, which induces genes responsible for cell survival. Additionally, activation of the Ras-ERK pathway further prevents programmed cell death. Elevated IL-5 levels disrupt the natural turnover of eosinophils, allowing them to accumulate in tissues and form the characteristic microabscesses. Therefore, therapeutic targeting of the IL-5 axis eliminates the essential survival signal required to maintain the chronic inflammatory lesions [27].

**Figure 2:**
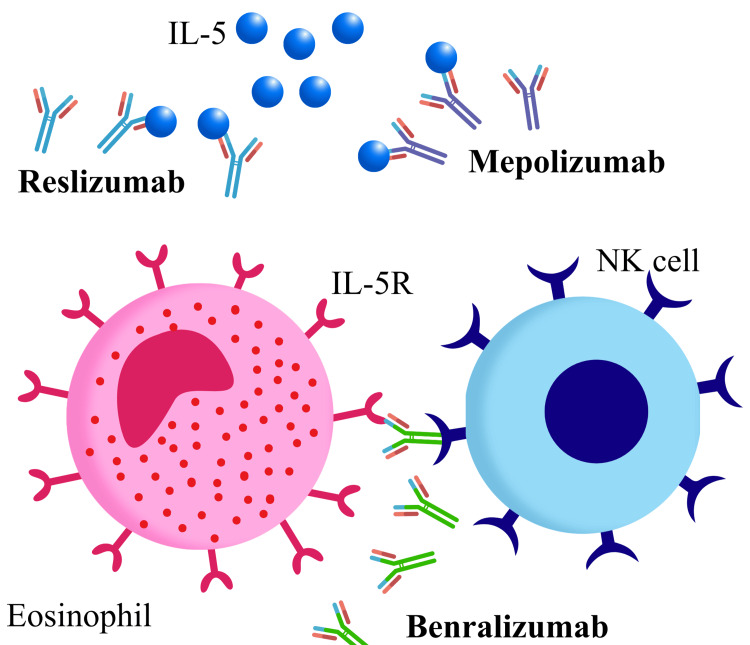
Mechanism of action of anti-IL-5 agents NK: natural killer; IL-5: interleukin-5; IL-5R: interleukin-5 receptor. Figure credits: Authors using Canva (Canva Pty Ltd, Surry Hills, New South Wales, Australia).

The available anti-IL-5 therapies work through specific mechanisms. Mepolizumab and reslizumab differ from benralizumab by targeting the IL-5 ligand itself, which prevents the cytokine from interacting with the receptor on the eosinophil surface. In contrast, benralizumab binds to the alpha subunit of the IL-5 receptor. This specific binding recruits natural killer cells to induce eosinophil apoptosis through antibody-dependent cell-mediated cytotoxicity, resulting in a more rapid and near-complete depletion of eosinophils compared to ligand-blocking agents, as shown in Figure 2 [28].

Targeting IL-5 or its receptor has proven effective in various eosinophil-associated disorders beyond KD. Although reslizumab has not been explicitly reported in the reviewed KD cases, its established efficacy as an anti-IL-5 agent in related conditions is worth mentioning. In eosinophilic granulomatosis with polyangiitis (EGPA), mepolizumab significantly increased the accrued weeks of remission, as 28% of patients achieved remission for at least 24 weeks compared with 3% in the placebo group. Additionally, mepolizumab reduced the annualized relapse rate by 50% and facilitated glucocorticoid dose reduction [29]. In hypereosinophilic syndrome, mepolizumab treatment lowered the proportion of patients experiencing flares by 50% relative to placebo and improved fatigue scores [30]. Benralizumab showed efficacy in chronic rhinosinusitis with nasal polyps by significantly decreasing nasal polyp and nasal blockage scores, although it did not significantly delay the time to first nasal polyp surgery [31]. In patients with inadequately controlled asthma and elevated blood eosinophils, reslizumab reduced the frequency of clinical asthma exacerbations by 50-59% and improved lung function. These findings indicate the utility of targeting the IL-5 pathway in managing eosinophilic inflammation [32]. However, the therapeutic efficacy observed in related eosinophilic conditions such as EGPA should be applied to KD with caution, as differences in disease pathogenesis may influence treatment response.

Clinical management

Current evidence suggests a risk-stratified treatment algorithm to improve patient outcomes. While surgical excision remains the primary approach for diagnosis and initial treatment, monotherapy is recommended only for patients without significant risk factors. A recent systematic review identified four key prognostic predictors for recurrence: tumor diameter ≥3 cm, symptom duration ≥5 years, peripheral blood eosinophilia ≥20%, and serum IgE levels ≥10,000 IU/mL [9]. For patients exhibiting any of these high-risk characteristics, the optimal first-line approach is surgical excision combined with adjuvant therapy. This includes radiotherapy or systemic immunosuppression using corticosteroids or steroid-sparing agents (cyclosporine, mycophenolate mofetil, tacrolimus, and leflunomide) to reduce the high risk of relapse [26].

**Figure 3:**
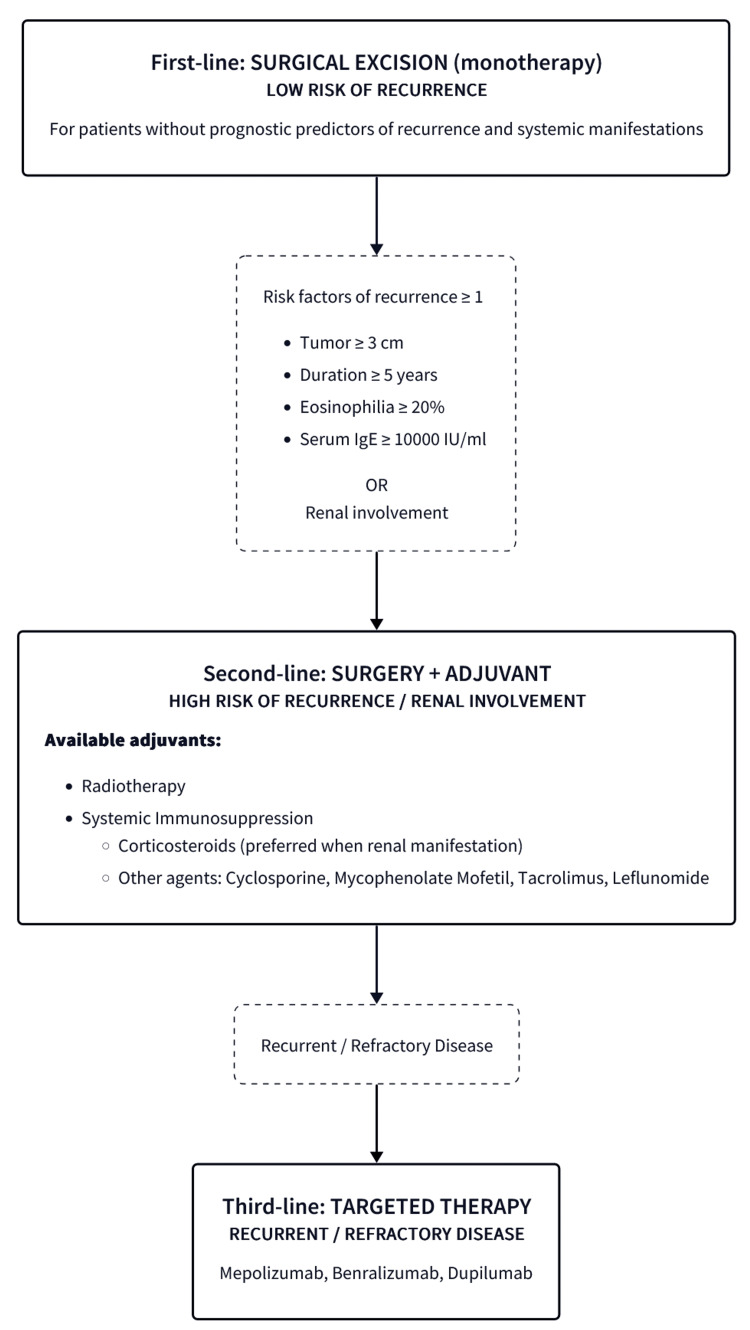
Proposed treatment algorithm for Kimura disease This algorithm is proposed by the authors and is based on interpretation of limited evidence. It is not derived from formal guideline recommendations or comparative clinical trials. It requires further validation and prospective clinical studies to confirm its efficacy. Figure credits: Authors using D2 Documentation (Terrastruct, San Francisco, CA, USA).

There is no established consensus for the management of KD. Therefore, we propose a stepwise therapeutic strategy shown in Figure 3. The first-line treatment consists of surgical excision for diagnostic confirmation and treatment of low-risk lesions. The second-line treatment involves combination therapy (surgery plus radiotherapy or systemic immunosuppression) for patients meeting the high-risk criteria of recurrence, those with renal involvement (where corticosteroids are preferred), and patients requiring steroid-sparing non-steroidal agents. The third-line treatment is reserved for targeted biologic therapies for recurrent, refractory, or steroid-intolerant cases [5,9,26]. This proposed algorithm is based on limited evidence and requires further validation and prospective studies to confirm its efficacy. 

Although no adverse events were documented in reports reviewed here, data from larger clinical trials provide a more comprehensive safety profile for anti-IL-5 agents. In the pooled analysis of reslizumab trials, it was reported that the drug was well tolerated over long-term treatment durations exceeding one year [33]. The incidence of adverse events in the reslizumab group was comparable to placebo, with the most frequent events being worsening asthma, nasopharyngitis, and upper respiratory tract infections. This analysis found no increased risk of malignancies or parasitic infections. While anaphylaxis remains a potential risk with intravenous biologics, such events were very rare in the study population [33]. Similarly, the long-term safety of benralizumab was confirmed. Frequency of adverse events did not increase with prolonged exposure up to two years [34]. The most common adverse events included viral upper respiratory tract infections (14-16%) and worsening asthma (7-10%). The study demonstrated that the safety profile observed during the initial randomized phases was maintained during the extension phase, with no new safety signals emerging over time [34]. In patients with EGPA treated with mepolizumab, it was noted that adverse events were reported at similar rates in the treatment and placebo groups (97% vs. 94%) [29]. However, specific local injection-site reactions occurred more frequently with mepolizumab (15%) compared to placebo (13%). Systemic reactions, including hypersensitivity, were also slightly more common in the mepolizumab group (6%) than in the placebo group (1%). Despite these findings, the overall rate of serious adverse events was comparable between the two groups, supporting a favorable risk-benefit ratio even in patients with systemic vasculitis [29].

Limitations

This review is primarily limited by its reliance on case reports and small case series (n = 9 studies, 11 patients). The small sample size is a direct result of the condition's rarity and the limited number of published cases, rather than strict inclusion criteria. Such a study design introduces selection bias and prevents the performance of a quantitative meta-analysis to calculate precise efficacy rates. The absence of control groups, combined with the concurrent use of corticosteroids and immunosuppressants in many cases, complicates the assessment of the biologic agents' independent therapeutic effects. The off-label use of these interventions resulted in significant heterogeneity regarding dosage and treatment duration, which ranged from three months to four years. Finally, although the search strategy covered anti-IL-5 agents broadly, no studies using reslizumab were identified, and conclusions are restricted to mepolizumab and benralizumab. As prospective randomized controlled trials may be difficult to conduct due to the rarity of this disease, multicenter prospective registries or international collaborative cohorts are required to validate these findings and standardize dosing regimens.

## Conclusions

Anti-IL-5 agents, specifically mepolizumab and benralizumab, are a potential therapeutic option for the management of refractory or recurrent KD. Currently, mepolizumab is supported by a higher number of documented CRs, while benralizumab serves as an alternative. In our cohort, no direct head-to-head comparison was performed, and it remains unknown which one is superior. Given the very limited sample size of our benralizumab cohort (n = 3), it is not possible to compare the efficacy to mepolizumab. These agents have demonstrated efficacy in reducing tumor burden and controlling eosinophilia, serving as a steroid-sparing option for patients who experience high recurrence rates or significant morbidity from conventional therapies. There is insufficient evidence supporting biologic therapy as a first-line treatment, but it is considered a third-line option, specifically for patients with recurrent, refractory, or steroid-intolerant disease. While the observed safety profile is favorable with no specific drug-related adverse events reported in the analyzed cohort, the interpretation of these results requires caution due to significant methodological constraints. The current evidence base is limited to small, heterogeneous case reports and lacks control groups, complicating the assessment of the biologics' independent therapeutic effects. Consequently, findings are limited by the small cohort size (n = 11), and the potential publication bias requires caution when interpreting the reported efficacy. Therefore, until prospective randomized controlled trials are conducted to validate efficacy and establish standardized dosing regimens, anti-IL-5 therapy should be regarded as an off-label intervention reserved only for carefully selected cases.

## Appendices

Appendix 1: Complete search strategy for each database

SCOPUS

TITLE-ABS-KEY ("Kimura disease" OR "Kimura's" OR "Kimura") AND TITLE-ABS-KEY (Mepolizumab" OR "Reslizumab" OR "Benralizumab" OR "Anti-IL5" OR "anti-IL-5" OR "IL-5 receptor")

EMBASE

("Kimura disease" OR “Kimuras” OR "Kimura") AND ("Mepolizumab" OR "Reslizumab" OR "Benralizumab" OR "Anti-IL5" OR "IL-5 receptor")

Performing this search required deleting the apostrophe in “Kimura’s” to run the search. Search used “Kimuras” instead.

PUBMED

("Kimura disease" OR "Kimura's" OR "Kimura") AND ("Mepolizumab" OR "Reslizumab" OR "Benralizumab" OR "Anti-IL5" OR "IL-5 receptor")

Appendix 2: Risk-of-bias assessment using Joanna Briggs Institute (JBI) critical appraisal tools for case reports

**Table 2:** Risk-of-bias assessment using Joanna Briggs Institute (JBI) critical appraisal tools for case reports Q1-Q8 indicate questions 1 to 8 based on the JBI risk assessment tool for case reports [15]. The risk of bias was considered high if the study achieved up to 49% of "yes" scores, moderate if it scored between 50% and 75% of "yes" scores, and low with more than 75% of "yes" scores.

Author	Q1	Q2	Q3	Q4	Q5	Q6	Q7	Q8	Sum of yes	Risk of bias level
Ho et al. [17]	Y	N	Y	Y	Y	Y	N	Y	6	Moderate
Kinoshita et al. [18]	Y	N	Y	Y	Y	Y	N	Y	6	Moderate
Al Shammari et al. [19]	Y	N	Y	Y	Y	Y	N	Y	6	Moderate
Tao et al. [20]	Y	N	Y	Y	Y	Y	Y	Y	7	Low
Du et al. [22]	Y	N	Y	Y	Y	Y	N	Y	6	Moderate
Yoon et al. [23]	Y	Y	Y	Y	Y	Y	Y	Y	8	Low
Szeto et al. [24]	Y	N	Y	Y	Y	Y	N	Y	6	Low

Appendix 3: Risk-of-bias assessment using Joanna Briggs Institute (JBI) critical appraisal tools for case series

**Table 3:** Risk-of-bias assessment using Joanna Briggs Institute (JBI) critical appraisal tools for case series Q1-Q10 indicate questions 1 to 10 based on the JBI risk assessment tool for case series [16]. The risk of bias was considered high if the study achieved up to 49% of "yes" scores, moderate if it scored between 50% and 75% of "yes" scores, and low with more than 75% of "yes" scores.

Author	Q1	Q2	Q3	Q4	Q5	Q6	Q7	Q8	Q9	Q10	Sum of yes	Risk of bias level
Zhu et al. [21]	Y	Y	Y	Y	Y	Y	Y	Y	Y	Y	10	Low
Talmon et al. [25]	Y	Y	Y	Y	Y	Y	Y	Y	Y	Y	10	Low
